# Development and validation of an AI approach for hyperbilirubinemia assessment from routine haematology in high-altitude settings

**DOI:** 10.7189/jogh.16.04347

**Published:** 2026-10-30

**Authors:** Yulong Liu, Mao Huang, Juanjuan Mou, Dexi Yongzong, Bo Lin, Xuelian Zhou, Fan Li, Jin Li, Ruoying Huang

**Affiliations:** 1 Department of Laboratory Medicine, The Affiliated Dazu’s Hospital of Chongqing Medical University, Chongqing, China; 2 College of Artificial Intelligence Medicine, Chongqing Medical University, Chongqing, China; 3 Department of Blood Transfusion, The Affiliated Dazu’s Hospital of Chongqing Medical University, Chongqing, China; 4 Department of Blood Transfusion, The People’s Hospital of Chaya County, Tibet, China

**Keywords:** hyperbilirubinemia, high-altitude hypoxia, machine learning, haematological parameters, risk stratification

## Abstract

**Background:**

Hypobaric hypoxia at extreme altitudes induces compensatory erythropoiesis, alters bilirubin metabolism, and increases the risk of high-altitude hyperbilirubinemia (HBIL). Conventional serum bilirubin screening in these regions is frequently constrained by limited medical infrastructure. This study aims to develop a machine learning pipeline that uses universally accessible routine haematological parameters to infer serum bilirubin status and stratify disease severity.

**Methods:**

We conducted a retrospective cohort study of permanent high-altitude residents (>2,900 m) to develop and validate diagnostic models using routine haematological parameters. We employed a Tabular Prior-Data Fitted Network for primary diagnosis and clinical severity stratification (latent *vs.* overt jaundice). We evaluated model performance using the area under the receiver operating characteristic curve (AUROC) and validated the approach in an independent temporal hold-out validation cohort. We interpreted feature importance using SHapley Additive exPlanations (SHAP).

**Results:**

The model exhibited strong discriminative capacity, achieving an AUROC of 0.918 for primary diagnosis and 0.888 for disease severity stratification in the internal validation set. It maintained predictive efficacy in the temporal hold-out validation cohort, with AUROC values of 0.923 and 0.905, respectively. Interpretability analysis revealed that the magnitude of compensatory erythrocytosis (Hb and Hct) was primarily associated with the initial onset of HBIL. Furthermore, erythrocyte morphological heterogeneity (RDW-SD) distinguished the transition to overt jaundice.

**Conclusions:**

This study establishes a scalable, artificial intelligence-driven framework for screening and risk-stratifying high-altitude HBIL using routine blood parameters. This approach provides a practical, low-cost strategy for proactive clinical management in resource-constrained extreme environments.

Bilirubin, once regarded as an inert end product of heme catabolism, is now recognised as a pleiotropic mediator of oxidative stress, immune regulation, and metabolic homeostasis [1,2]. Mildly elevated bilirubin confers systemic protection against cardiovascular diseases [3–5], metabolic syndrome [6–8], and certain malignancies [9–12]. Conversely, excessive unconjugated bilirubin exhibits severe neurotoxicity [13,14].

High-altitude environments (>2,500 m) represent a unique physiological extreme. Prolonged hypobaric hypoxia triggers profound compensatory erythropoiesis, elevating red cell mass and turnover [15,16]. As bilirubin is derived from heme catabolism, this adaptation reshapes baseline bilirubin metabolism [17–19]. Yet conventional serum bilirubin testing in these regions is constrained by inadequate laboratory infrastructure and logistical barriers.

Routine hematological parameters are intrinsically coupled with bilirubin synthesis and remain universally accessible, even in the most resource-limited settings. However, inferring bilirubin status from these parameters alone has remained elusive [20,21]. Although recent advances in artificial intelligence (AI) offer promising capabilities in deciphering nonlinear biological associations [22–25], their application to bilirubin surveillance in extreme environments remains underexplored.

To address this critical diagnostic gap, we developed a Tabular Prior-Data Fitted Network (TabPFN) model to identify hyperbilirubinemia (HBIL) and stratify its clinical severity (latent *vs.* overt jaundice) using routine blood parameters. We profiled a Tibetan cohort permanently residing above 2,900 m, capturing the hematological signatures of chronic hypobaric hypoxia and their relationship to bilirubin elevation across two sequential clinical tasks.

This study establishes a low-cost, AI-driven screening strategy that extends bilirubin surveillance to primary-care settings where conventional biochemical testing is unavailable. By decoupling HBIL risk assessment from specialised laboratory infrastructure, this approach lays a foundation for proactive health management and early disease detection in resource-limited, extreme environments.

## METHODS

### Study design and high-altitude cohort assembly

This retrospective cohort study examined the association between routine haematological parameters and HBIL under high-altitude conditions. Data were extracted from the electronic health records (EHR) and laboratory information system (LIS) of the Physical Examination Center, Chaya County People's Hospital, Tibet, China (altitude range = 2,900–5,000 m; x̄ = 3,500 m). The hospital serves over 76,000 predominantly indigenous permanent high-altitude residents (Figure S1 in the **Online Supplementary Document**).

The derivation cohort comprised individuals examined between January 2022 and December 2024; a temporal hold-out validation cohort was drawn from the same centre (January to June 2025). Eligibility was restricted to permanent residents (verified by national identity card) to ensure haematological indices reflected long-term high-altitude adaptation. Genotype data were unavailable in this retrospective EHR-based study. Complete liver function tests were available for all participants; none exhibited biochemical evidence of hepatocellular injury or predominantly conjugated HBIL. Individuals with hepatic or biliary disease, haemolytic conditions, or non-altitude aetiologies of HBIL were excluded. Medical history records were available for review.

The study adhered to the Declaration of Helsinki and was approved by the Medical Ethics Committee of the Affiliated Dazu's Hospital of Chongqing Medical University (Approval No. 2024039). Informed consent was waived given the retrospective use of de-identified data.

### Clinical definitions and outcomes

We defined two sequential clinical tasks: HBIL diagnosis and risk stratification. HBIL was defined as serum total bilirubin (TBIL) > 17.1 μmol/L, per the National Clinical Laboratory Operation Procedures. Patients with TBIL 17.1–34.2 μmol/L were classified as latent jaundice (this range may reflect benign adaptation or early pathology; periodic reassessment is prudent). TBIL > 34.2 μmol/L was classified as overt jaundice, warranting prompt clinical evaluation.

### Data preprocessing and feature engineering

From 4,710 eligible individuals, we identified 630 HBIL patients and 4,080 healthy controls. To mitigate class imbalance, we randomly undersampled controls to create a 1:1 analytical cohort (n = 1,260). Validation metrics from this balanced set are for model comparison only and do not reflect real-world prevalence. The cohort was partitioned into a training set (80%, n = 1008) and an internal validation set (20%, n = 252) by stratified random sampling. The risk-stratification subset comprised 510 training cases (372 latent, 138 overt jaundice), 120 internal validation cases (108 latent, 12 overt), and 48 temporal hold-out cases (36 latent, 12 overt).

To reduce collinearity among the 26 haematological parameters, we applied LASSO regression to the Step 1 training set, with the optimal λ determined by 10-fold cross-validation. This retained 22 predictors, which served as the unified input for both Step 1 and Step 2, ensuring methodological consistency while avoiding selection bias from independent feature selection given the limited overt jaundice cases in Step 2.

### Machine learning pipeline and model development

We compared logistic regression (LR), support vector machine (SVM), random forest (RF), XGBoost (XGB), CatBoost (CATB), and the TabPFN. Because conventional deep learning models are prone to overfitting on small-to-medium tabular cohorts, we incorporated TabPFN. TabPFN is pre-trained on synthetic structural causal models (SCMs) and performs Bayesian inference *via* in-context learning without data set-specific training. It assumes that real-world tabular data fall within the prior distribution of its pre-training SCMs. This assumption aligns with the well-characterised causal hierarchies of hematopoietic parameters. Our training set ( ~ 1,000 samples, 22 features) falls at the boundary of the validated limits (≤1,000 samples, ≤100 features). Its performance is known to degrade when these bounds are exceeded. All baseline models underwent hyperparameter optimisation *via* grid search with 10-fold cross-validation; TabPFN required no tuning.

### Model evaluation and interpretability

The performance of both diagnostic and risk-stratification models was assessed using a held-out internal validation set. The area under the receiver operating characteristic curve (AUROC) served as the primary metric for predictive discrimination, supported by accuracy, precision (positive predictive value), recall (sensitivity), and the F1-score. To evaluate statistical robustness, 95% confidence intervals (CIs) for these metrics were generated through empirical bootstrapping with 2,000 iterations.

To decipher the feature dependencies of the optimal model, we incorporated SHapley Additive exPlanations (SHAP) analysis. By quantifying the marginal contribution of each haematological parameter, SHAP provides both global feature importance and local prediction interpretability. This analytical layer validates the biological plausibility of the predictions against known high-altitude physiological adaptations, thereby substantiating the model's potential utility in clinical decision support.

### Statistical analysis

Continuous variables are presented as median with interquartile range (IQR) and were analysed using the Mann-Whitney U test, following normality assessment. Categorical variables are reported as counts (percentages) and were evaluated using the χ^2^ test. A two-sided *P* < 0.05 was considered statistically significant. All statistical and computational analyses were performed using *R*, version 4.4.1 (R Foundation for Statistical Computing, Vienna, Austria) and Python, version 3.11.9 (Python Software Foundation, Wilmington, DE, USA), incorporating the Scikit-learn, SciPy, version 1.7.1 (SciPy community), and TabPFN, version 2.5_default (Prior Laboratories GmbH, Freiburg im Breisgau, Germany) libraries. Model comparisons were performed using DeLong's test for paired receiver operating characteristic curves, with Holm correction for multiple pairwise comparisons. Adjusted *P* < 0.05 was considered statistically significant.

## RESULTS

### Baseline characteristics

The analytical cohort comprised 1260 permanent high-altitude residents – 630 HBIL and 630 controls (Table S1 in the **Online Supplementary Document**). Consistent with hypobaric hypoxic adaptation, the HBIL group exhibited significantly elevated erythropoietic indices – specifically red blood cell (RBC) counts, haemoglobin (Hb), and haematocrit (Hct) – compared to healthy controls (all *P* < 0.001). Concurrently, patients with HBIL demonstrated reduced platelet (PLT) counts and decreased lymphocyte abundance (all *P* < 0.001), suggesting potential crosstalk between amplified heme metabolism and immune modulation. Demographically, the HBIL group was significantly older and displayed a distinct sex distribution (both *P* < 0.001).

To evaluate risk-specific haematological features, the HBIL cohort was stratified into latent jaundice (n = 480) and overt jaundice (n = 150) subgroups (Table S2 in the **Online Supplementary Document**). Although overall erythropoietic parameters were elevated across the pan-HBIL population, the overt jaundice subgroup exhibited a relative decrease in RBC, Hb, and Hct (all *P* < 0.05). Conversely, disease progression to overt jaundice was characterised by increased erythrocyte morphological heterogeneity (elevated RDW-SD and RDW-CV) and a substantial reduction in PLT counts (all *P* < 0.001). Furthermore, the overt jaundice group was significantly older (*P* < 0.001), whereas the sex distribution remained comparable between the risk strata (*P* = 0.974).

### Feature selection and dimensionality reduction

To identify discriminative predictors from the 26 haematological parameters, we applied LASSO regression to the training cohort. We varied the *L*_1_ regularisation penalty (**Figure 1**, Panel A) and selected the optimal penalisation parameter (log(λ) = −4.07) at the minimum cross-validated mean squared error (**Figure 1**, Panel B), which retained 22 features. The choice of λ_min_ reflects a prediction-oriented strategy that prioritises retention of informative features over maximal parsimony. The correlation network (**Figure 1****,** Panel C) revealed physiologically coupled clusters across the erythroid and leukocyte lineages. UMAP projection of the 22-dimensional feature space (**Figure 1**, Panel D) displayed substantial spatial overlap and nonlinear entanglement between the healthy and HBIL groups, with no definitive linear decision boundary. This topological complexity justifies deploying high-capacity architectures such as TabPFN.

**Figure 1:**
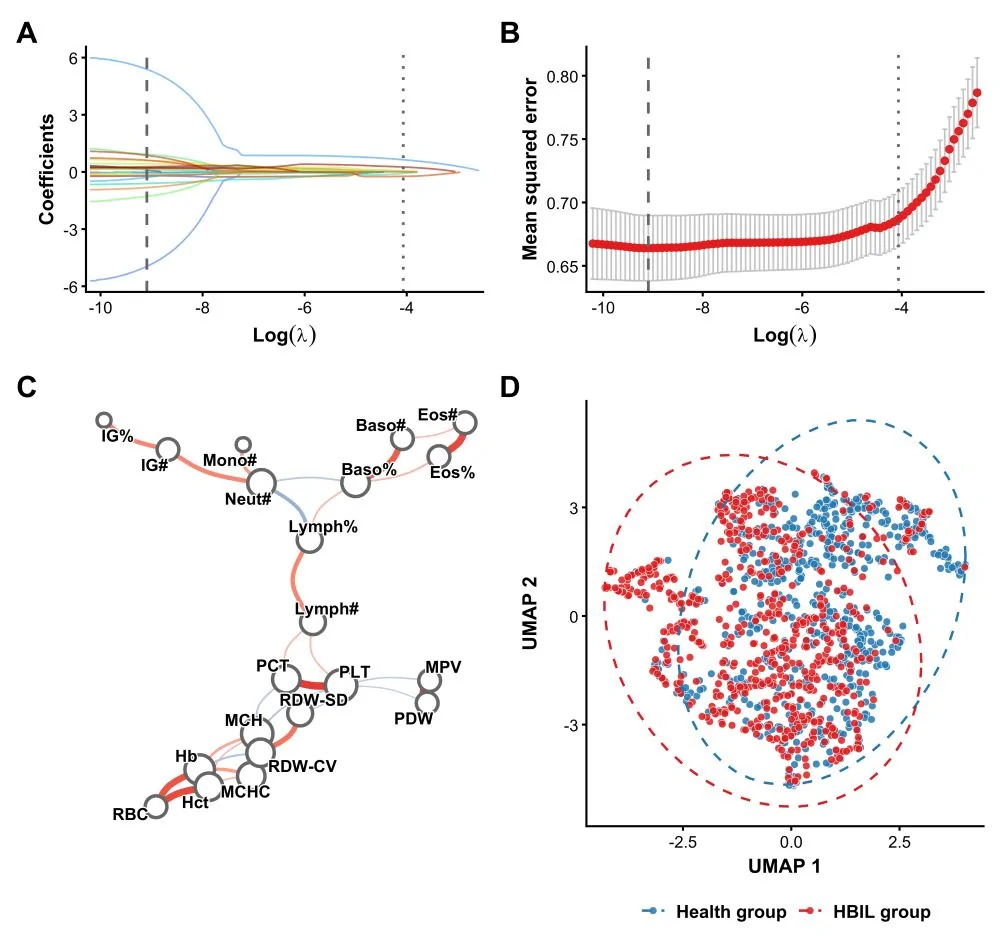
Feature selection and dimensionality reduction of haematological profiles. Panel A. LASSO coefficient paths. The trajectories illustrate the shrinkage of the 26 initial haematological parameters as the *L*_1_ regularisation penalty (log(λ)) increases. Panel B. Cross-validation for optimal parameter selection. The mean squared error (MSE) curve is derived from 10-fold cross-validation. The dashed vertical line indicates the optimal log(λ) value, which corresponds to the selection of 22 features. Panel C. Feature correlation network. Network visualisation of the 22 retained variables, displaying physiological correlations across the erythroid, leukocyte, and thrombocyte lineages. Panel D. UMAP projection. Two-dimensional Uniform Manifold Approximation and Projection (UMAP) of the clinical cohort based on the refined 22-feature space, illustrating the nonlinear spatial distribution and overlap between the healthy (blue) and HBIL (red) groups. Baso# – absolute basophil count, Baso% – basophil percentage, Eos# – absolute eosinophil count, Eos% – eosinophil percentage, Hb – haemoglobin, HBIL – hyperbilirubinemia, Hct – haematocrit, IG# – absolute immature granulocyte count, IG% – immature granulocyte percentage, LASSO – least absolute shrinkage and selection operator, Lymph# – absolute lymphocyte count, Lymph% – lymphocyte percentage, MCH – mean corpuscular haemoglobin, MCHC – mean corpuscular haemoglobin concentration, Mono# – absolute monocyte count, MSE – mean squared error, MPV – mean platelet volume, Neut# – absolute neutrophil count, PCT – plateletcrit, PDW – platelet distribution width, PLT – platelet count, RBC – red blood cell count, RDW-CV – red cell distribution width coefficient of variation, RDW-SD – red cell distribution width standard deviation, UMAP – uniform manifold approximation and projection.

### Model performance in HBIL screening

We evaluated the diagnostic performance of all models on the held-out internal validation cohort. Linear baselines (LR and SVM) achieved AUROCs of 0.853 and 0.850, respectively (**Figure 2**, Panel A, **Table 1**). Ensemble methods, particularly CATB (AUROC = 0.910) and RF (AUROC = 0.895), captured nonlinear interactions more effectively and significantly outperformed the linear models.

**Figure 2:**
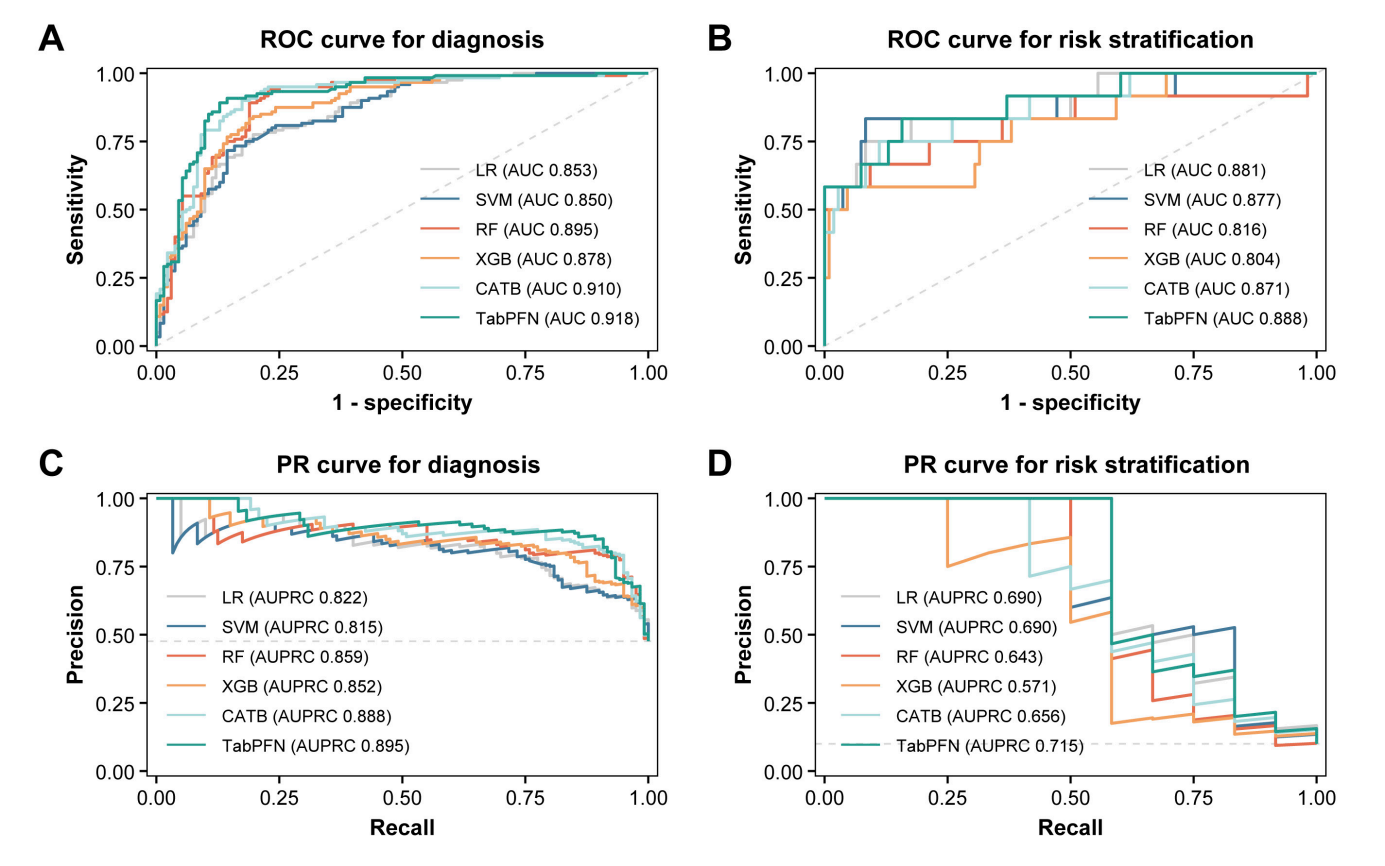
Predictive performance of the machine learning pipeline for HBIL diagnosis and risk stratification. Panels A and C. Primary HBIL diagnosis. Receiver operating characteristic (ROC) and precision-recall (PR) curves evaluating model performance on the held-out validation cohort. TabPFN achieved the highest AUROC (0.918) and AUPRC (0.895), numerically exceeding tree-based ensembles and linear baselines. Panels B and D. Clinical risk stratification. ROC and PR curves for distinguishing latent from overt jaundice. Linear baselines (LR, SVM) showed improved relative performance compared with the diagnostic task. TabPFN yielded the highest numerical AUROC (0.888) and AUPRC (0.715). AUC – area under the curve, AUPRC – area under the precision-recall curve, AUROC – area under the receiver operating characteristic curve, CATB – CatBoost, HBIL – hyperbilirubinemia, LR – logistic regression, PR – precision–recall, RF – random forest, ROC – receiver operating characteristic, SVM – support vector machine, TabPFN – Tabular Prior-Data Fitted Network, XGB – extreme gradient boosting.

**Table 1:** Predictive performance of machine learning models for HBIL diagnosis and risk stratification in the internal validation cohort

	Model	AUROC	Accuracy	Precision	Recall	F1-score
**Step 1**	LR	0.853 ± 0.023	0.782 ± 0.026	0.788 ± 0.039	0.742 ± 0.040	0.764 ± 0.031
	SVM	0.850 ± 0.024	0.778 ± 0.026	0.771 ± 0.039	0.758 ± 0.038	0.764 ± 0.030
	RF	0.895 ± 0.021	0.802 ± 0.025	0.792 ± 0.037	0.791 ± 0.038	0.791 ± 0.029
	XGB	0.878 ± 0.022	0.806 ± 0.025	0.809 ± 0.036	0.775 ± 0.038	0.791 ± 0.030
	CATB	0.910 ± 0.019	0.841 ± 0.024	0.850 ± 0.034	0.808 ± 0.037	0.828 ± 0.027
	TabPFN	0.918 ± 0.019	0.865 ± 0.022	0.858 ± 0.032	0.858 ± 0.033	0.858 ± 0.025
**Step 2**	LR	0.881 ± 0.060	0.900 ± 0.027	0.496 ± 0.136	0.585 ± 0.148	0.527 ± 0.121
	SVM	0.877 ± 0.070	0.908 ± 0.026	0.535 ± 0.141	0.585 ± 0.148	0.548 ± 0.123
	RF	0.816 ± 0.089	0.900 ± 0.028	0.499 ± 0.140	0.585 ± 0.148	0.528 ± 0.123
	XGB	0.804 ± 0.076	0.909 ± 0.025	0.540 ± 0.140	0.585 ± 0.148	0.551 ± 0.123
	CATB	0.871 ± 0.061	0.917 ± 0.025	0.583 ± 0.149	0.585 ± 0.148	0.573 ± 0.127
	TabPFN	0.888 ± 0.058	0.934 ± 0.023	0.698 ± 0.149	0.585 ± 0.148	0.625 ± 0.126

AUROC – area under the receiver operating characteristic curve, CATB – CatBoost, HBIL – hyperbilirubinemia, LR – logistic regression, RF – random forest, SVM – support vector machine, TabPFN – Tabular Prior-Data Fitted Network, XGB – extreme gradient boosting

TabPFN achieved the highest AUROC (0.918) for HBIL diagnosis (**Figure 2****,** Panel A, **Table 1**). DeLong tests with Holm correction showed that TabPFN significantly outperformed LR, SVM, RF, and XGB (all *P* < 0.05) as presented in Table S3 in the **Online Supplementary Document**. The comparison with CATB did not reach significance (ΔAUROC = 0.008, DeLong *P* = 0.239), indicating comparable discriminative performance between the two top models. CATB required systematic grid-search hyperparameter optimisation within a 10-fold cross-validation framework. TabPFN achieved comparable performance without data set-specific tuning, a property of its pre-trained in-context learning mechanism that offers practical advantages for clinical reproducibility and deployment. In precision-recall analysis (**Figure 2**, Panel C), TabPFN yielded an area under the precision-recall curve (AUPRC) of 0.895, numerically exceeding CATB (0.888) and other baselines.

### Model performance in risk stratification

In the risk-stratification task, model performance shifted relative to the diagnosis task (**Figure 2**, Panel B). Random forest and XGBoost, which performed well in diagnosis, declined to AUROCs of 0.816 and 0.804, respectively (**Table 1**). Conversely, LR reached 0.881 and SVM 0.877, both exceeding the ensemble architectures. This inversion may reflect the small overt jaundice subgroup (approximately 150 cases), which predisposes ensemble models to overfit noise in the minority class. Alternatively, the haematological signature of disease progression may be intrinsically simpler than that of initial diagnosis. This possibility is consistent with the SHAP analysis (**Figure 3**, Panel E), which revealed a predominantly linear relationship between RDW-CV and severity risk. We refrain from drawing strong conclusions from this observation alone. The limited sample size yields high variance in performance estimates, and several factors may contribute to the observed pattern.

**Figure 3:**
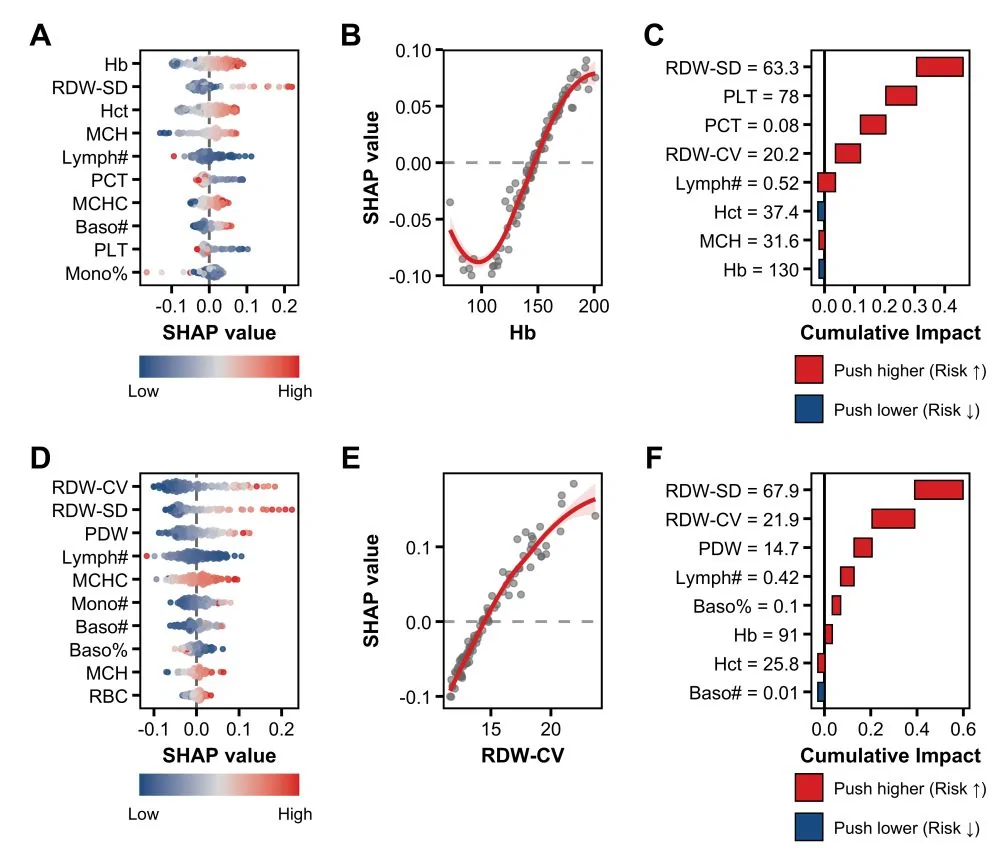
Mechanistic interpretability of the TabPFN model *via* SHAP analysis. Panels A–C. Primary HBIL diagnosis. Panel A. Global SHAP summary plot. Parameters reflecting the magnitude of compensatory erythrocytosis (Hb, Hct) and erythrocyte morphological heterogeneity (RDW-SD) are identified as the principal predictive features. Panel B. SHAP dependence plot for Hb. The curve exhibits a nonlinear relationship with an inflection point, indicating a physiological threshold beyond which hepatic bilirubin clearance is likely compromised. Panel C. Local SHAP waterfall plot illustrating individualised feature contributions for a representative patient's diagnostic prediction. Panels D–F. Clinical risk stratification. Panel D. Global SHAP summary plot for distinguishing latent from overt jaundice. The feature importance hierarchy is altered compared to the diagnostic task, with indicators of erythrocyte morphological heterogeneity (RDW-CV, RDW-SD) and altered platelet indices (PDW) emerging as the primary determinants of disease severity. Panel E. SHAP dependence plot for RDW-CV, illustrating a near-linear positive association with the probability of overt jaundice. Panel F. Local SHAP waterfall plot demonstrating the cumulative contribution of haematological parameters to risk stratification for an individual patient. Baso# – absolute basophil count, Baso% – basophil percentage, Hb – haemoglobin, HBIL – hyperbilirubinemia, Hct – haematocrit, Lymph# – absolute lymphocyte count, MCH – mean corpuscular haemoglobin, MCHC – mean corpuscular haemoglobin concentration, Mono# – absolute monocyte count, Mono% – monocyte percentage, PCT – plateletcrit, PLT – platelet count, PDW – platelet distribution width, RBC – red blood cell count, RDW-CV – red cell distribution width coefficient of variation, RDW-SD – red cell distribution width standard deviation, SHAP – SHapley Additive exPlanations, TabPFN – Tabular Prior-Data Fitted Network.

TabPFN yielded the highest numerical AUROC of 0.888 (**Table 1**) in the risk-stratification task. In precision-recall analysis (**Figure 2**, Panel D), it achieved an AUPRC of 0.715, exceeding the linear baselines (LR and SVM, both 0.690). DeLong tests did not reach significance for any pairwise comparison – all Holm-corrected *P* > 0.05 (Table S3 in the **Online Supplementary Document**), likely reflecting the limited power of the small overt jaundice subgroup. Precision (0.698) and F1-score (0.625) indicate a non-negligible false-positive rate. The discordance between AUROC and precision reflects the low prevalence of overt jaundice (approximately 23.8% of the HBIL cohort), a recognised effect of class imbalance. These metrics underscore that the risk-stratification model should be understood as a preliminary screening aid prompting confirmatory testing, rather than a standalone diagnostic tool.

### Temporal hold-out validation

To assess the robustness of the pipeline, we evaluated performance on a temporal hold-out validation cohort from a non-overlapping period, from January to June 2025 (**Table 2**). In the HBIL diagnosis task, TabPFN achieved a temporal hold-out AUROC of 0.923, numerically exceeding tree-based ensembles (CATB = 0.910, XGB = 0.914) and linear baselines. TabPFN also achieved a recall of 0.917. High sensitivity supports its use as a screening tool in resource-limited settings, where minimising false negatives is critical.

**Table 2:** Predictive performance of machine learning models in the independent temporal hold-out validation cohort

	Model	AUROC	Accuracy	Precision	Recall	F1-score
**Step 1**	LR	0.869 ± 0.032	0.789 ± 0.037	0.704 ± 0.061	0.834 ± 0.054	0.762 ± 0.046
	SVM	0.867 ± 0.032	0.763 ± 0.038	0.668 ± 0.061	0.833 ± 0.054	0.740 ± 0.046
	RF	0.904 ± 0.026	0.797 ± 0.036	0.701 ± 0.059	0.877 ± 0.047	0.777 ± 0.043
	XGB	0.914 ± 0.025	0.824 ± 0.034	0.740 ± 0.058	0.877 ± 0.047	0.801 ± 0.041
	CATB	0.910 ± 0.026	0.832 ± 0.033	0.762 ± 0.058	0.854 ± 0.050	0.804 ± 0.042
	TabPFN	0.923 ± 0.023	0.857 ± 0.031	0.775 ± 0.055	0.917 ± 0.039	0.839 ± 0.038
**Step 2**	LR	0.812 ± 0.066	0.753 ± 0.062	0.503 ± 0.120	0.750 ± 0.131	0.594 ± 0.110
	SVM	0.817 ± 0.064	0.753 ± 0.062	0.503 ± 0.120	0.750 ± 0.131	0.594 ± 0.110
	RF	0.805 ± 0.087	0.773 ± 0.060	0.532 ± 0.123	0.750 ± 0.131	0.614 ± 0.110
	XGB	0.839 ± 0.076	0.896 ± 0.044	0.817 ± 0.121	0.750 ± 0.131	0.774 ± 0.103
	CATB	0.827 ± 0.067	0.794 ± 0.058	0.566 ± 0.127	0.750 ± 0.131	0.637 ± 0.111
	TabPFN	0.905 ± 0.056	0.794 ± 0.058	0.566 ± 0.127	0.750 ± 0.131	0.637 ± 0.111

AUROC – area under the receiver operating characteristic curve, CATB – CatBoost, LR – logistic regression, RF – random forest, SVM – support vector machine, TabPFN – Tabular Prior-Data Fitted Network, XGB – extreme gradient boosting

In the risk-stratification task, TabPFN yielded the highest numerical AUROC (0.905 ± 0.056). The relatively large standard error reflects the small number of overt jaundice cases (n = 12) in this cohort and the consequent statistical uncertainty in the point estimate. The rank order of model performance was broadly consistent with that observed in the internal validation set. Given the small case count, these performance estimates should be interpreted with caution. The risk-stratification model is best regarded as a preliminary screening aid rather than a definitive clinical decision tool.

### Mechanistic interpretability *via* SHAP analysis

We computed SHA*P* values to identify features driving TabPFN predictions and to assess their biological plausibility. Given known collinearity within erythroid and platelet clusters (**Figure 1**, Panel C), SHAP importance is interpreted at the level of physiological axes – erythroid mass, erythrocyte morphological heterogeneity, and platelet dynamics – rather than as precise individual feature rankings.

For HBIL diagnosis (**Figure 3**, Panel A), the principal predictors reflected compensatory erythrocytosis (Hb and Hct) and erythrocyte morphological heterogeneity (RDW-SD). The SHAP dependence plot for Hb (**Figure 3**, Panel B) revealed a sigmoidal relationship with a distinct inflection point beyond which HBIL risk increased sharply, consistent with a physiological compensatory threshold beyond which heme catabolism may overwhelm hepatic bilirubin clearance.

The feature importance hierarchy shifted in the risk-stratification task (**Figure 3**, Panel D). While absolute erythroid mass drove the diagnostic classification, indicators of erythrocyte morphological heterogeneity and turnover – RDW-CV, RDW-SD, and platelet distribution width (PDW) – emerged as the principal determinants for distinguishing latent from overt jaundice. Elevated RDW-CV exhibited a near-linear positive correlation with disease severity risk (**Figure 3**, Panel E), suggesting that progressive anisocytosis and altered platelet dynamics mark a transition from compensatory erythropoiesis to haematological decompensation. Decreased absolute lymphocyte counts were also associated with higher risk.

Local SHAP waterfall plots (**Figure 3**, Panels C and F) illustrated the directional contribution of each parameter at the individual patient level, supporting the model's potential utility for individualised risk assessment.

### Clinical implementation of the AI TibetMedLab platform

The AI TibetMedLab platform integrates with the existing LIS/EHR infrastructure at Chaya County People's Hospital. Upon release of haematology analyser results, the platform automatically extracts the 22-feature panel, executes TabPFN inference, and appends an AI-generated report to the patient record. This workflow is designed to enable opportunistic HBIL screening regardless of the clinical indication.

End-to-end execution on standard hospital workstations ranged from eight to 12 seconds per sample. DeepSeek-R1, accessed *via* API, converts the multidimensional risk scores and SHAP-derived feature contributions into natural-language clinical reports (Figures S2 and S3 and Supplemental Note 1 in the **Online Supplementary Document**). Standardised training covering platform operation, risk-label interpretation, and follow-up procedures can be completed in approximately 1.5 hours.

## DISCUSSION

This study establishes a machine learning pipeline that infers HBIL status and stratifies severity using routine haematological parameters, extending bilirubin surveillance to settings where biochemical testing is unavailable.

The clinical rationale lies in the healthcare realities of the Qinghai-Tibet Plateau. Basic haematology analysers are widely deployed at primary-care facilities, whereas biochemistry platforms required for serum bilirubin measurement remain concentrated at county-level hospitals. A 2024 national policy mandating biochemistry analyser deployment to key township hospitals by 2025 underscores that such capacity is not yet broadly available. Moreover, biochemistry analysers require reliable power, cold-chain reagent storage, and skilled personnel, all of which are frequently compromised by extreme weather and logistical constraints above 3,500 m. Even where available, sample batching and geographic dispersion can delay results beyond clinical utility. Machine learning inference is therefore not a replacement for serum bilirubin testing, but a triage tool deployable immediately using existing infrastructure, enabling frontline clinicians to identify patients needing urgent referral while monitoring those with latent jaundice on site.

The model's performance reflects the coupling between compensatory erythropoiesis and heme catabolism. SHAP analysis revealed a sigmoidal dependence of HBIL risk on haemoglobin concentration. Chronic high-altitude exposure induces sustained erythropoiesis to maintain tissue oxygenation; the resulting expansion of erythroid mass increases the substrate load for heme catabolism. This is consistent with a physiological threshold beyond which heme degradation overwhelms hepatic bilirubin clearance [26].

For risk stratification, the feature importance hierarchy shifted: progression from latent to overt jaundice was driven by erythrocyte morphological heterogeneity (RDW-CV, RDW-SD) and platelet dynamics (PDW, PLT) rather than absolute erythroid mass. Elevated RDW and reduced platelet counts are consistent with accelerated erythrocyte senescence, oxidative membrane damage, and splenic sequestration [27]. Lower lymphocyte counts implicate systemic inflammatory stress. These findings suggest a transition from stable hypoxic adaptation to haematological decompensation marked by deteriorating erythrocyte quality and turnover. Previous studies have documented links among hypoxia-inducible factor pathway activation, polycythemia, and altered metabolic states [27,28], but have not translated these haematological shifts into a functional screening tool.

For HBIL diagnosis, TabPFN achieved performance comparable to CATB, the best tree-based ensemble (ΔAUROC = 0.008, DeLong *P* = 0.239), and significantly outperformed LR, SVM, RF and XGB (all Holm-corrected *P* < 0.05). Critically, TabPFN attained this performance without data set-specific hyperparameter tuning. This no-tuning property – stemming from its pre-trained in-context learning mechanism – reduces dependence on machine learning engineering expertise, a practical advantage in resource-constrained settings. Its contribution is one of convenience and robustness, not a statistically significant improvement in AUROC over the best tree-based alternative. In the risk-stratification task, no pairwise model comparison reached significance. We refrain from drawing strong conclusions from this observation: the limited number of overt jaundice cases (approximately 150) yields high variance in performance estimates, and the haematological signature of disease severity may be intrinsically simpler than that of initial diagnosis, consistent with the predominantly linear SHAP dependence observed for RDW-CV.

Several considerations qualify the reported metrics. These metrics derive from a 1:1 balanced set, reflecting 50% prevalence. At real-world lower prevalence, negative predictive value (NPV) rises and precision declines, while AUROC and sensitivity remain prevalence-independent. The higher NPV strengthens the model’s rule-out capability, consistent with its screening role. The decline in precision reinforces that positive results should prompt confirmatory testing – which uses the same blood draw and is available at county-hospital level – rather than standalone clinical action.

Mild bilirubin elevation in the latent jaundice range (TBIL = 17.1–34.2 μmol/L) may reflect benign adaptive HBIL, consistent with the established antioxidant and cardioprotective properties of bilirubin [29], rather than incipient disease. The term denotes a diagnostically indeterminate state, not a disease requiring treatment, and a single blood panel cannot reliably distinguish benign adaptation from early pathology. The clinical utility of risk stratification therefore lies in identifying individuals warranting confirmatory testing and periodic monitoring, not in labelling them as diseased.

To translate these findings into practice, we developed the AI TibetMedLab platform. The platform integrates with existing LIS/EHR infrastructure to enable opportunistic HBIL screening and uses DeepSeek-R1 to generate interpretable natural-language clinical reports [30,31]. TabPFN performs inference in a single forward pass, completing each prediction in eight to 12 seconds on standard hospital workstations. By rendering the basis of each prediction transparent, this design addresses the interpretability gap that has historically limited AI adoption at the point of care. It thereby provides a scalable framework for proactive HBIL management in high-altitude settings.

Several limitations warrant consideration. First, this was a retrospective, single-centre study of ethnically homogeneous Tibetan residents. The temporal hold-out cohort was drawn from the same institution and population and therefore provides evidence of temporal validity only, not external generalisability. High-altitude populations globally harbour distinct adaptive haplotypes [27], and the current model is calibrated for long-term indigenous Tibetan residents. Furthermore, the study relied on national identity card verification as a proxy for high-altitude ancestry, without genetic characterisation of adaptive variants (*e.g.* EPAS1, EGLN1) or individual residence-duration data. Its applicability to other ethnic groups, individuals of mixed ancestry, or recent migrants from lower altitudes therefore requires independent validation. Second, the risk-stratification task is constrained by the small number of overt jaundice cases, yielding substantial uncertainty in performance estimates. The validation cohort is insufficiently powered for subgroup equity analyses across sex, age, and haemoglobin strata. The model's reliance on erythropoietic markers raises the possibility of differential performance in individuals with physiologically lower haemoglobin, such as women of reproductive age, and elderly patients with attenuated hypoxic erythropoietic responses. Formal equity analyses across demographic strata should be a priority for future multi-centre studies. Third, SHAP-based feature importance can be unstable with collinear predictors; we therefore interpret results at the level of physiological axes rather than individual feature rankings. The training set falls at the boundary of the validated limits of TabPFN, yet the model assumes that real-world haematological data share the causal structure of its synthetic pre-training prior. Whether this assumption generalises to other clinical domains remains to be evaluated. Congenital HBIL (*e.g.* Gilbert's syndrome) cannot be excluded without genetic testing, though it typically produces mild unconjugated HBIL unlikely to affect the risk-stratification task anchored at TBIL > 34.2 μmol/L. Multi-centre external validation across geographically distinct settings with diverse genetic ancestries and analyser platforms is needed to establish generalisability and recalibrate decision boundaries.

## CONCLUSIONS

In summary, this study translates haematological signatures of high-altitude hypoxia into an AI-driven, accessible screening tool for HBIL. By leveraging universally available routine blood parameters and a model architecture that requires no data set-specific tuning, this approach reduces dependence on resource-intensive clinical infrastructure. It extends proactive bilirubin surveillance to primary-care settings and offers a practical framework for clinical decision-making in extreme environments. Generalisability to other high-altitude populations with distinct genetic ancestries and different analyser platforms remains to be established through multi-centre external validation.

## Additional material


Online Supplementary Document

## Data Availability

**Data availability:** The data sets generated and analysed during the current study are not publicly available due to patient privacy regulations and restrictions imposed by the ethics committee. The data contain sensitive patient information from electronic health records. However, de-identified data are available from the corresponding author on reasonable request and with permission of the Chaya County People's Hospital.
