# Supplementary material for: Development and validation of an AI approach for hyperbilirubinemia assessment from routine haematology in high-altitude settings
Source: J Glob Health. 2026 Oct 30;16:04347. doi: 10.7189/jogh.16.04347 (PMC13721646; doi:10.7189/jogh.16.04347)
Supplement: Supplementary file 1 [file jogh-16-04347-s001.pdf]

Supplement to: Liu Y, Huang M, Mou J, Dexi Y, Lin B, Zhou X, Li F, Li J, Huang R. Development and validation of an AI approach for hyperbilirubinemia assessment from routine haematology in high-altitude settings. J Glob Health. 2026;16:04347.

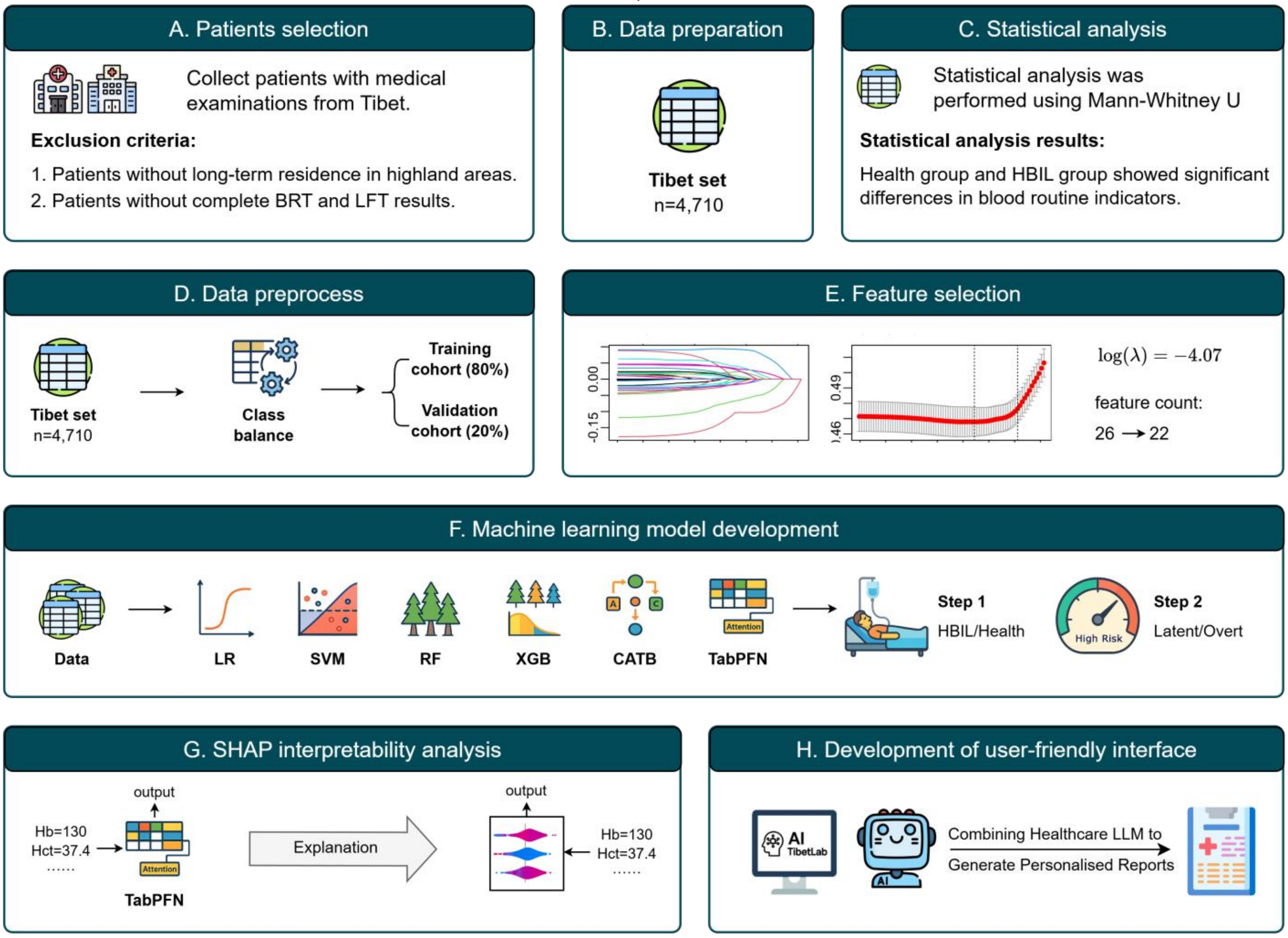

Figure S1. **Analytical workflow for the machine learning-driven diagnosis and clinical management of HBIL.**

(A) Flowchart detailing patient selection, outlining inclusion criteria for clinical cohort assembly in Tibet and exclusion criteria (non-permanent high-altitude residents and incomplete routine blood or liver function test profiles).

(B) Assembly of the final analytical cohort (Tibet cohort, n = 4,710).

(C) Statistical comparison of baseline clinical characteristics between the healthy control and HBIL groups (Mann-Whitney U test, $P < 0.05$).

(D) Data preprocessing pipeline, including class balancing and cohort partitioning into training (80%) and validation (20%) sets.

(E) Feature selection via least absolute shrinkage and selection operator (LASSO) regression . The plot depicts the cross-validated mean squared error (MSE) against , with the optimal penalty ( ) distilling 22 critical features from the initial 26.

(F) Systematic benchmarking of representative machine learning architectures (LR, SVM, RF, XGB, CATB, and TabPFN) across a sequential clinical workflow: Step 1 (primary HBIL diagnosis) and Step 2 (risk stratification).

(G and H) Mechanistic interpretability and clinical translation. Global and local SHapley Additive exPlanations (SHAP) analyses elucidate the decision boundaries of the TabPFN model. The predictive engine is subsequently integrated with a domain-specific large language model (LLM) to establish the Ai TibetMedLab platform, facilitating the automated generation of interpretable clinical reports for resource-constrained settings.

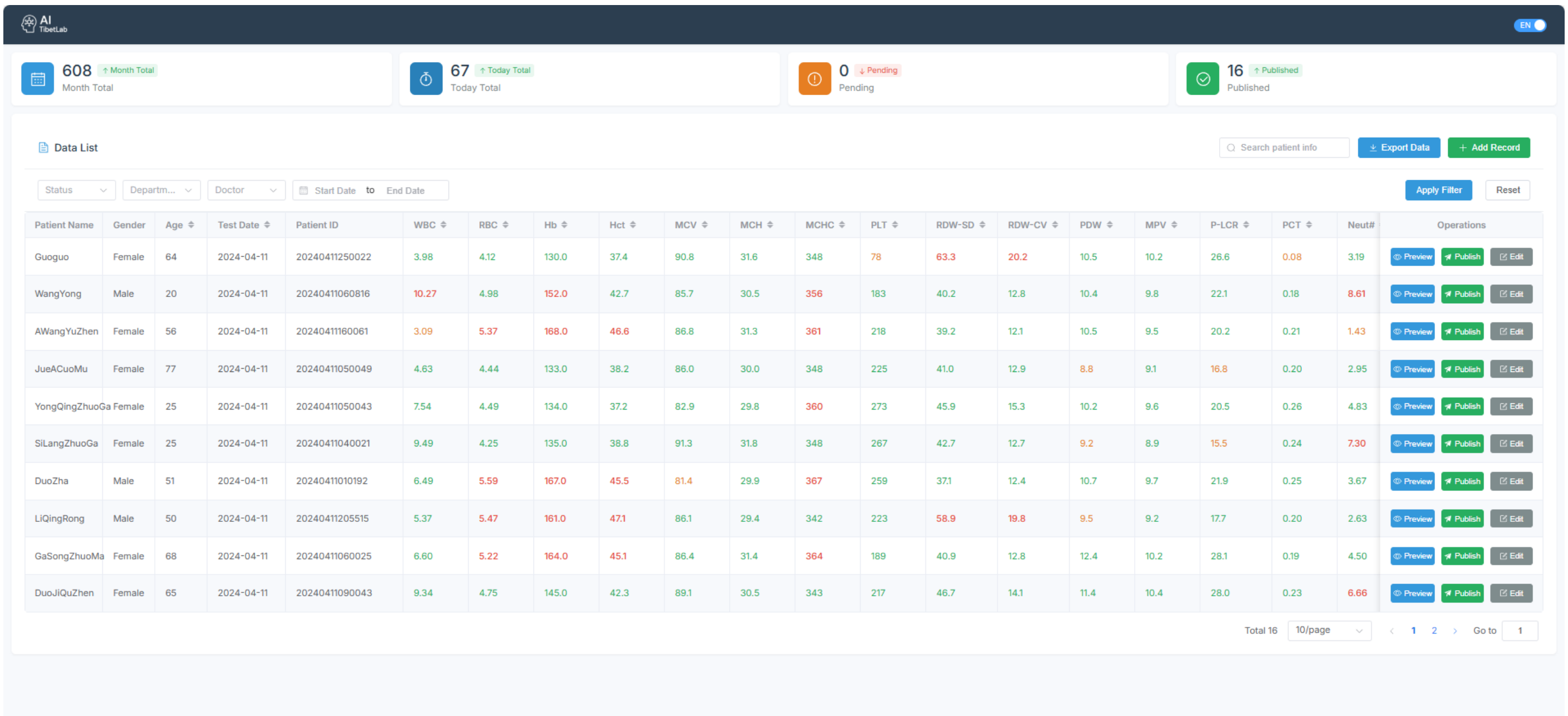

Figure S2. Ai TibetMedLab system interface: patient data list view. This figure displays the main dashboard of the Ai TibetMedLab system, showcasing a tabular list of patient data. Each patient entry has 'Preview', 'Publish', and 'Edit' options under 'Operations'. Abnormal blood parameter values are highlighted.

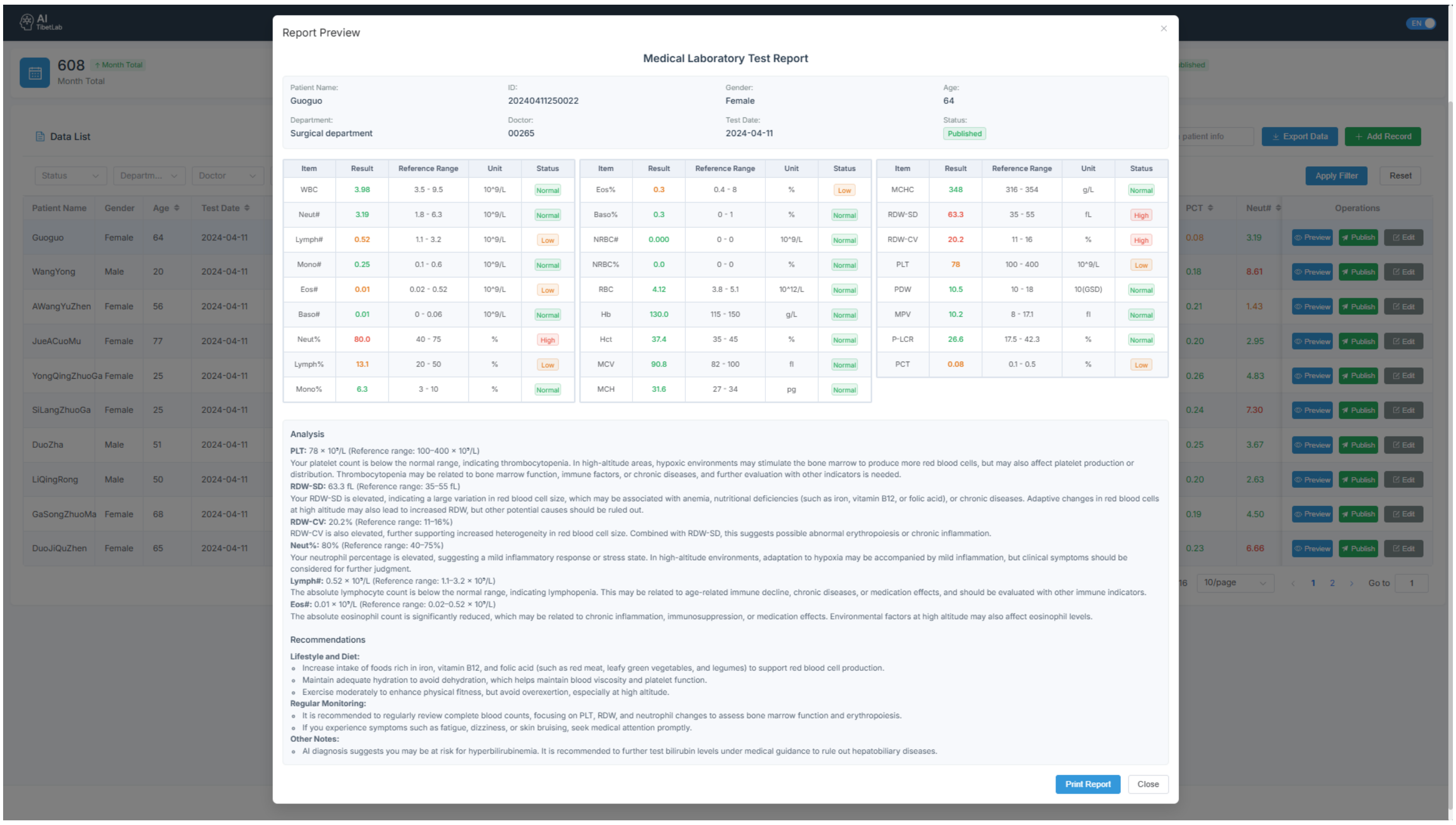

Figure S3. Ai TibetMedLab system interface: medical laboratory test report view. This figure illustrates an example of a generated "Medical Laboratory Test Report" within the Ai TibetMedLab system for a patient named "Guoguo." The top section displays patient demographics (Name, ID, Department, Division, Test Date, Age, Gender, Status). The main body lists individual blood test parameters (e.g., WBC, Lymph#, Neut#, Hb, Hct, PLT, RDW-CV) with their 'Item' name, 'Result', 'Reference Range', 'Unit', 'Limit', and 'Status' (indicating normal or abnormal, e.g., "Low", "High"). Below the test results, an "Analysis" section provides an automated interpretation of abnormal findings, linking them to potential physiological conditions (e.g., high-altitude adaptation, anemia, inflammation) and explaining the significance of parameters like PLT, RDW-SD, RDW-CV, Neutrophil percentage, and Lymphocyte percentage. A "Recommendations" section offers lifestyle and dietary advice (e.g., iron-rich foods, hydration, regular monitoring) and guidance on when to seek further medical attention, including a note about potential hyperbilirubinemia risk.

Table S1. Baseline demographic and clinical characteristics of the HBIL and healthy control groups.

	Overall	Health group	HBIL group	*P*-Value
**n**	1260	630	630	
**Age**	36.000 [27.750,55.000]	33.000 [25.000,49.000]	39.000 [31.000,58.000]	
**Gender**	680 (53.968)	435 (69.048)	245 (38.889)	<0.001
	580 (46.032)	195 (30.952)	385 (61.111)	
**RBC**	4.960 [4.380,5.420]	4.720 [4.250,5.180]	5.155 [4.613,5.598]	<0.001
**Hb**	147.000 [127.000,164.000]	137.000 [118.000,154.000]	157.000 [139.000,172.000]	<0.001
**Hct**	42.500 [37.275,47.200]	40.000 [35.025,44.475]	44.800 [40.425,49.300]	<0.001
**MCH**	30.100 [28.500,31.400]	29.500 [27.400,30.800]	30.600 [29.325,31.800]	<0.001
**MCHC**	344.000 [336.000,352.000]	342.000 [332.000,349.000]	347.000 [340.000,354.000]	<0.001
**PLT**	225.000 [179.000,276.000]	237.000 [198.000,291.000]	212.000 [157.000,260.750]	<0.001
**RDW-SD**	42.900 [40.300,46.500]	42.800 [40.300,45.200]	42.900 [40.300,48.200]	0.002
**RDW-CV**	13.500 [12.700,14.800]	13.600 [12.800,14.600]	13.300 [12.600,15.000]	0.085
**PDW**	10.700 [9.600,12.400]	10.400 [9.300,12.000]	11.000 [9.800,12.700]	<0.001
**MPV**	9.800 [9.200,10.500]	9.700 [9.100,10.400]	9.900 [9.300,10.700]	<0.001
**PCT**	0.220 [0.180,0.270]	0.230 [0.193,0.280]	0.210 [0.160,0.260]	<0.001
**Neut#**	4.100 [2.970,6.402]	4.185 [2.980,6.420]	4.010 [2.940,6.317]	0.677
**Lymph#**	1.615 [1.140,2.163]	1.665 [1.232,2.230]	1.560 [1.040,2.098]	<0.001
**Mono#**	0.420 [0.320,0.570]	0.410 [0.320,0.568]	0.430 [0.340,0.570]	0.080
**Eos#**	0.060 [0.020,0.110]	0.060 [0.020,0.107]	0.060 [0.020,0.110]	0.753
**Baso#**	0.030 [0.020,0.040]	0.020 [0.020,0.040]	0.030 [0.020,0.040]	0.016
**Lymph%**	25.800 [15.700,35.300]	26.500 [17.400,36.000]	25.200 [14.400,34.500]	0.004
**Mono%**	6.300 [5.100,7.800]	6.200 [4.800,7.600]	6.500 [5.400,8.000]	<0.001
**Eos%**	0.900 [0.300,1.900]	0.900 [0.300,1.700]	0.900 [0.200,2.000]	0.673
**Baso%**	0.400 [0.200,0.600]	0.400 [0.200,0.600]	0.400 [0.200,0.700]	0.005
**IG#**	0.010	0.010	0.010	0.826

	[0.010,0.030]	[0.010,0.030]	[0.010,0.030]	
**IG%**	0.200	0.200	0.200	0.546
	[0.100,0.300]	[0.100,0.300]	[0.100,0.400]	
**TBIL**	17.080	8.610	26.020	<0.001
	[8.615,26.005]	[6.213,11.255]	[22.370,33.730]	

Table S2. Baseline demographic and clinical characteristics of the latent jaundice and overt jaundice groups.

	Overall	Health group	HBIL group	*P*-Value
**n**	630	480	150	
**Age**	39.000	37.000	53.000	
	[31.000,58.000]	[30.000,57.000]	[34.000,64.000]	
**Gender**	245	186	59	0.974
	(38.889)	(38.750)	(39.333)	
	385	294	91	
	(61.111)	(61.250)	(60.667)	
**RBC**	5.155	5.205	4.945	0.005
	[4.613,5.598]	[4.680,5.613]	[4.268,5.545]	
**Hb**	157.000	158.000	149.000	0.012
	[139.000,172.000]	[142.000,172.250]	[134.000,168.000]	
**Hct**	44.800	45.400	43.200	0.010
	[40.425,49.300]	[41.175,49.300]	[38.425,49.250]	
**MCH**	30.600	30.500	31.050	0.002
	[29.325,31.800]	[29.200,31.625]	[29.725,32.375]	
**MCHC**	347.000	347.000	348.000	0.412
	[340.000,354.000]	[339.000,354.000]	[340.000,356.000]	
**PLT**	212.000	221.000	167.000	<0.001
	[157.000,260.750]	[171.000,269.250]	[109.000,225.250]	
**RDW-SD**	42.900	42.500	47.600	<0.001
	[40.300,48.200]	[40.075,46.500]	[41.325,55.925]	
**RDW-CV**	13.300	13.100	14.550	<0.001
	[12.600,15.000]	[12.500,14.300]	[13.000,17.375]	
**PDW**	11.000	10.900	11.600	0.002
	[9.800,12.700]	[9.800,12.400]	[10.100,13.500]	
**MPV**	9.900	9.800	10.100	0.001
	[9.300,10.700]	[9.300,10.600]	[9.500,11.000]	
**PCT**	0.210	0.220	0.170	<0.001
	[0.160,0.260]	[0.170,0.260]	[0.110,0.240]	
**Neut#**	4.010	3.870	4.430	0.182
	[2.940,6.317]	[2.908,6.312]	[3.042,6.357]	
**Lymph#**	1.560	1.680	1.175	<0.001
	[1.040,2.098]	[1.177,2.183]	[0.712,1.655]	
**Mono#**	0.430	0.420	0.460	0.154
	[0.340,0.570]	[0.330,0.570]	[0.350,0.607]	
**Eos#**	0.060	0.060	0.040	<0.001
	[0.020,0.110]	[0.020,0.120]	[0.010,0.090]	
**Baso#**	0.030	0.030	0.020	0.003
	[0.020,0.040]	[0.020,0.040]	[0.010,0.037]	
**Lymph%**	25.200	27.000	19.050	<0.001
	[14.400,34.500]	[15.400,35.425]	[9.250,28.325]	
**Mono%**	6.500	6.300	7.000	0.017
	[5.400,8.000]	[5.400,7.600]	[5.425,8.975]	
**Eos%**	0.900	1.100	0.600	0.001
	[0.200,2.000]	[0.300,2.100]	[0.100,1.800]	
**Baso%**	0.400	0.400	0.300	0.014

	[0.200,0.700]	[0.275,0.700]	[0.200,0.600]	
**IG#**	0.010	0.010	0.020	0.151
	[0.010,0.030]	[0.010,0.030]	[0.010,0.030]	
**IG%**	0.200	0.200	0.200	0.057
	[0.100,0.400]	[0.100,0.300]	[0.200,0.400]	

Table S3. Pairwise DeLong test results for model comparison in the internal validation cohort.

Step	Comparison	AUROC (95% CI)		ΔAUROC	DeLong p	Adjusted p
	vs. TabPFN	**TabPFN**	**Other model**			
**Step 1**	**LR**	**0.918±0.019**	**0.853±0.023**	**0.065***	**<0.001***	**<0.001***
	**SVM**	**0.918±0.019**	**0.850±0.024**	**0.068***	**<0.001***	**<0.001***
	**RF**	**0.918±0.019**	**0.895±0.021**	**0.023***	**0.024***	**0.049***
	**XGB**	**0.918±0.019**	**0.878±0.022**	**0.040***	**0.006***	**0.017***
	**CATB**	0.918±0.019	0.910±0.019	0.008	0.239	0.239
**Step 2**	**LR**	0.888±0.058	0.881±0.060	0.007	0.765	1.000
	**SVM**	0.888±0.058	0.877±0.070	0.011	0.749	1.000
	**RF**	0.888±0.058	0.816±0.089	0.072	0.049	0.244
	**XGB**	0.888±0.058	0.804±0.076	0.084	0.105	0.421
	**CATB**	0.888±0.058	0.871±0.061	0.017	0.208	0.624

Supplemental Note 1. Template and prompt for AI Generation of Patient Blood Report Analysis.
**You are a doctor who is writing a detailed interpretation and recommendations for a patient's medical report. **

Task: Review the patient's blood routine report results. Write a professional, rigorous and informative report section for the patient, directly addressing the patient (using "you", "your"). Please use standard medical abbreviations to refer to blood parameters.
The report section should contain two main parts:
1. **Analysis:** Detailed description of the meaning and potential impact of each specific abnormal result.
2. **Recommendations:** Based on the analysis results and AI diagnostic prompts, provide lifestyle, diet and follow-up recommendations.
**The overall output should be concise and focused, only interpret abnormal indicators, do not give recommendations on the next step of examination and treatment, and any medical-related recommendations must be issued by professional doctors. **

Patient background information to be considered:
* Patient gender: {gender}
* Patient age: {age}
* **Special circumstances: {additional_information}**
* **AI diagnostic: {tabpfn_output_result}**

Reference ranges (for your reference - not listed in patient reports; abbreviations are consistent with patient result format):
```json
{
"WBC": { "min": 3.5, "max": 9.5, "unit": "10^9/L" },
"RBC": { "min": 3.8, "max": 5.1, "unit": "10^12/L" },
"Hb": { "min": 115, "max": 150, "unit": "g/L" },
"Hct": { "min": 35, "max": 45, "unit": "%" },
"PLT": { "min": 100.0, "max": 400.0, "unit": "10^9/L" },
"MCV": { "min": 82.0, "max": 100.0, "unit": "fl" },
"MCH": { "min": 27.0, "max": 34.0, "unit": "pg" },
"MCHC": { "min": 316, "max": 354, "unit": "g/L" },
"RDW-CV": { "min": 11, "max": 16, "unit": "%" },
"RDW-SD": { "min": 35, "max": 55, "unit": "fL" },
"Neut%": { "min": 40, "max": 75, "unit": "%" },
"Lymph#": { "min": 1.1, "max": 3.2, "unit": "10^9/L" },
"Lymph%": { "min": 20, "max": 50, "unit": "%" },
"Eos#": { "min": 0.02, "max": 0.52, "unit": "10^9/L" },
"Eos%": { "min": 0.4, "max": 8.0, "unit": "%" },
"NRBC#": { "min": 0, "max": 0, "unit": "10^9/L" },
"NRBC%": { "min": 0, "max": 0, "unit": "%" },
"PCT": { "min": 0.1, "max": 0.5, "unit": "%" }
}
```

Patient test results (please use these exact abbreviations and values):
```json
{
"Sex": "{gender}",
```

"Age": "{age}",
"WBC": "{wbc_value}",
"RBC": "{rbc_value}",
"Hb": "{hb_value}",
"Hct": "{hct_value}",
"MCV": "{mcv_value}",
"MCH": "{mch_value}",
"MCHC": "{mchc_value}",
"PLT": "{plt_value}",
"RDW-SD": "{rdw_sd_value}",
"RDW-CV": "{rdw_cv_value}",
"Neut%": "{neut_percent_value}",
"Lymph#": "{lymph_absolute_value}",
"Lymph%": "{lymph_percent_value}",
"Eos#": "{eos_absolute_value}",
"Eos%": "{eos_percent_value}",
"NRBC#": "{nrbc_absolute_value}",
"NRBC%": "{nrbc_percent_value}",
"PCT": "{pct_value}"
}
